# Severity of respiratory tract infections depends on the infectious dose. Perspectives for the next pandemic

**DOI:** 10.3389/fpubh.2024.1391719

**Published:** 2024-04-30

**Authors:** Kåre Mølbak, Thorkild I. A. Sørensen, Samir Bhatt, Frederik Plesner Lyngse, Lone Simonsen, Peter Aaby

**Affiliations:** ^1^Department of Veterinary and Animal Sciences, Faculty of Health and Medical Sciences, University of Copenhagen, Copenhagen, Denmark; ^2^Statens Serum Institut, Copenhagen, Denmark; ^3^Department of Public Health, Faculty of Health and Medical Sciences, University of Copenhagen, Copenhagen, Denmark; ^4^PandemiX Center, Roskilde University, Roskilde, Denmark; ^5^Department of Clinical Research, University of Southern Denmark, Odense, Denmark; ^6^Bandim Health Project, Bissau, Guinea-Bissau

**Keywords:** SARS-CoV-2, dose response, mathematical model, pandemic planning, respiratory infections

## Introduction

During the COVID-19 pandemic, much has been said about the importance of host-specific and virus-specific factors as predictors of the risk of infection and severity of disease. For example, host factors such as increased age, male gender, ethnicity, and comorbidities such as metabolic and pulmonary disorders have been recognized as risk factors for severe disease, whereas host immunity stemming from prior infection or vaccination against severe acute respiratory syndrome coronavirus 2 (SARS-CoV-2) is associated with reduced severity. Similarly, the viral evolution of SARS-CoV-2 over the past 4 years has been scrutinized to estimate changes in the relative transmissibility, virulence and vaccine-match of each emerging variant over time during the COVID-19 pandemic. However, despite this scrutiny, variation in transmissibility and severity of disease remain imperfectly understood.

**Figure 1:**
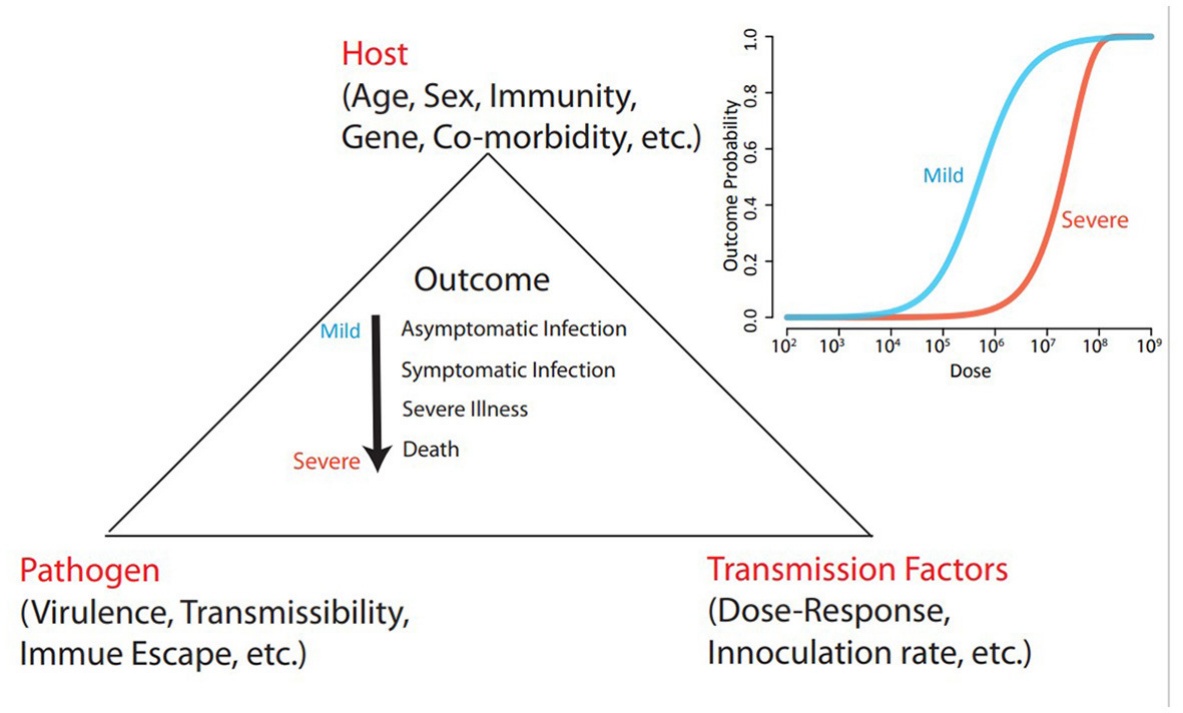
A model of interacting host, pathogen and transmission factors affecting the outcome of exposure to an infectious pathogen. The outcome is conceptualized as a continuum ranging from no infection over asymptomatic infection to severe illness or death. We hypothesize that the risk of a severe outcome depends on the dose of exposure. This hypothesis may explain incompletely understood variations in transmissibility and severity, and may be a key in improving preparations for the next pandemic. The inserted graph illustrates the principle of different dose-reponse curves for mild and severe outcome, and is based on salmonella-data from Teunis et al. (2).

An aspect that has been comparatively ignored is the importance of transmission factors such as the size of viral inoculation and the duration of exposure, i.e., the dose of exposure (see Figure 1). The dose of exposure is determined by human behavior, environmental conditions and mitigation strategies, such as indoor versus outdoor exposure, indoor crowding, indoor air ventilation and physical distancing. As observed for a range of pathogens, the risk of getting infected, and in some studies, also the disease severity and post infection sequelae depend on the dose encountered (1–10). In 2021, Van Damme et al. (11) postulated that the dose of SARS-CoV-2 at infection was an important missing factor in understanding several incompletely explained observations in the epidemiology of COVID-19. Nevertheless, epidemiological models (and common thinking) continue to parameterize exposure as a dichotomous phenomenon, where the susceptible host is being considered as either exposed (and at risk of infection and severe disease) or unexposed (and therefore not at risk). We hypothesize that a quantitative exposure approach, where dose of exposure is included as a factor that determine important factors such as risk of infection, incubation period, outcome of infection and transmissibility, may be helpful for our understanding of the epidemiology of COVID-19, also in the ongoing transition of the pandemic to endemicity. But more importantly, if this hypothesis can be generalized across other pathogens, a quantitative exposure approach to infection epidemiology may open new options for mitigation of a future severe pandemic, Disease X, and point a way forward to a control strategy with a gentler impact on society.

## Is the dose of exposure important in terms of disease severity?

There are plausible biological explanations for the existence of a dose-response relation. When pathogenic microorganisms (virus, bacteria, fungi or parasites) enter the human body, they encounter a system of barriers mounted by the host. These include physical and chemical barriers as well as non-specific innate and specific adaptive immunological responses (12). For the infectious agent to gain a foothold in the host and establish an infection, at least one microorganism must overcome these host barriers. We propose that it is not only the risk of infection that can be explained by the dose-response framework, but also that the continuum of outcomes of the infection from subclinical to severe illness depends on the infectious dose. Possibly, the incubation period may also be seen as a function of the infectious dose, usually with a shorter incubation following a high dose of exposure, as suggested for very different infections including cholera (13), measles (6) and HIV (14). Finally, transmissibility, i.e., the ability to generate more cases, may also be affected. We hypothesize that intensive exposure from a primary case will result in a higher viral load in the secondary case, and that this in turn will lead to a higher risk of ongoing transmission and possibly superspreading. The severity of the primary case is also critical for understanding severity of subsequent cases, i.e., a severe primary case will transmit more virus and generate more severe secondary cases (6). In very large families, institutions, refugee camps or virgin soil outbreaks where there are several subsequent generations of cases this may generate an exponential increase in severity of the disease as has been shown for measles (15).

Empirical data from a wide range of infections support our proposal to widen the risk factor paradigm to also include dose dependency. For example, the risk of becoming ill after exposure to gastrointestinal pathogens such as cholera (1, 13), salmonella and cryptosporidium (2) is known to be dose dependent. Also, for HIV infection, a dose-response relation is well established (3, 14). Likewise, studies of several respiratory tract infections including influenza (5), measles (6, 7, 15), pneumococcal disease (8), and tuberculosis (9) confirm or corroborate that intensity and duration of exposure are critical to understanding the outcomes of infection, including severity. For example, in a Danish study of hospitalized measles cases, children infected at home by siblings and therefore exposed to high doses were at greater risk of dying than children infected outside the home (6).

Less is known about dose-response relations for coronavirus infections but studies of human coronavirus 229E (10) and SARS-CoV-2 (11, 16–18) corroborate the application more generally. This is furthermore in line with several observations of the importance of viral dose of SARS-CoV-2 for infectivity (19–22), the correlation between viral load and disease severity (23), and a recent study showing that transmission of SARS-CoV-2 increased with duration of exposure (24).

Given these studies, the evidence for dose-response affecting the severity of measles, another airborne disease, and the biological rationale explained above, we hypothesize that there is a dose-response relation for the effects of airborne infections in general. The dose represents the number of virus in the inhaled air, and response may include all the consequences of exposure, ranging from the risk of becoming infected, the length of the incubation period, subsequent contagiousness, and the probability of severe disease outcomes, late sequelae and death.

This hypothesis is compatible with studies that show that increased ventilation and the use of face masks offer some protection against COVID-19 (25–27). Whereas the results from these studies also can be interpreted as reduced likelihood of a none-or-all process, ventilation and face masks reduces the exposure dose, which may in turn reduce the risk of getting infected as well as reduce the severity of illness. Although the model proposed by Koelle et al. (27) does not support this possibility, an experimental inquiry into this topic is clearly warranted. To this end we need laboratory studies, including animal models such as those which was established for SARS-CoV (4). There is a further need for observational studies with a specific focus on the role of dose of exposure with disease severity, incubation period and further transmissibility as outcomes. Studies of household transmission or outbreaks can serve as data sources at this end. The role of infectious inoculum may also be addressed in studies of the impact of personal protection devices, indoor versus outdoor exposure, and in investigation of ventilation systems and air cleaners on modifying the outcome of infection.

Human challenge studies provide the most important evidence for dose-response relations (1, 2, 10, 13, 28). However, human challenge studies may be difficult to perform due to ethical concerns. These concerns are of course a particular issue with infections with a severe outcome, and in experiments where the dose of potential exposure is enhanced rather than reduced. Therefore, a dose-response assessment will often depend on a panel of studies with different methods, including observational studies.

Finally, for mathematical modeling, we suggest adding quantitative exposure parameterization (rather than a dichotomous variable) to existing models. For this purpose, inspiration may be gained from Quantitative Microbial Risk Assessment (QMRA). QMRA is a systematic approach to provide information to understand the nature of the potential effects from microbial exposure, and the dose-response assessment phase is an essential quantitative element of QMRA. It estimates the risk of a hazard (for example, infection, illness or death) given a known dose of exposure to a pathogen. QMRA was first proposed for use in the treatment of water in microbiological risk management in the 1990s, and represents a mainstream tool to determine the microbial safety of e.g., food and water. Teunis et al. (2) provide an example of how to bridge QMRA and epidemiological data, and Koelle et al. (27) is a recent example of a quantitative framework for understanding the relationship between (i) inoculum dose and the risk of infection and (ii) inoculum dose and the risk of developing severe disease. Future work should aim to integrate a dose-response framework into models of population transmission and burden of illness.

## The perspective for a “Disease X scenario”

The dose-response paradigm may have important ramifications for pandemic response. A new disease with pandemic potential—Disease X—comes with many “known unknowns.” An exploration of the dose-response relation and its relevance for severity represents one of the “known unknowns,” and may have profound implications for the mitigation strategy.

If early evidence (e.g., studies of outbreaks and clusters) of a new Disease X of public health importance does support a dose-response relationship, we suggest that it would be reasonable to include this relationship in the mathematical models that underpin control and mitigation strategies. Initially, this could be done unconditionally on the severity of the disease, in order to understand the spread of Disease X. Later it would be relevant to include severity as a dose-dependent outcome, if data support this extension. Modeling may be based first on observation of the patterns of the spread of the disease as it was during the SARS-CoV-2 initially, and then followed by assessment of the effects of various measures to reduce the exposure dose. If the disease is lethal or severe, investigating mechanisms for reducing the probability of infection, the severity of disease, and/or mortality is important.

If it is impossible to contain Disease X at the epicenter, and the pathogen is spreading globally, such control strategies will no longer have elimination as a goal. Rather, it will serve to limit the burden of disease until an effective treatment or vaccine is available. As we have experienced in the COVID-19 pandemic response, this comes with high societal costs due to the need to use of blunt measures (lockdowns, school closures and restrictions of movements). However, with compelling evidence of a dose dependency, efforts to control epidemic spread may also include environmental strategies to lower the infectious dose, a sort of “dilution strategy.” Such an effect could be achieved by meeting outdoors, avoiding exposure in overcrowded indoor settings and through increased mechanical ventilation indoor and the use of face masks.

The main objective in a public health response to a future Disease X is to minimize severe illness, reduce the burden on health facilities, minimize societal disruption, and preserve the economy of the society. We hypothesize that a focus on dose dependency of the emerging pathogen may be a key factor in designing future control strategies that achieve all that.

## Author contributions

KM: Conceptualization, Investigation, Writing – original draft, Writing – review & editing. TS: Conceptualization, Writing – original draft, Writing – review & editing. SB: Investigation, Methodology, Writing – original draft, Writing – review & editing. FL: Writing – original draft, Writing – review & editing. LS: Conceptualization, Writing – original draft, Writing – review & editing. PA: Conceptualization, Investigation, Methodology, Writing – original draft, Writing – review & editing.
